# Obstetrics

**Published:** 1912-03

**Authors:** D. C. Rayner


### OBSTETRICS.

**The use of Pituitrin in Obstetrics.**—The action of pituitrin on the uterus during labour has now been studied by numerous observers, and we are in a position to judge to some extent of its value in obstetric practice. The drug, as is well known, is obtained from the posterior lobe of the pituitary gland. Apart from its stimulating action on uterine muscle, pituitrin raises the blood-pressure, and increases the force of the contraction of the heart. It also increases diuresis, intestinal peristalsis, and the contraction of the bladder. In a series of thirty-four cases in which the drug was employed during labour, it was found that its action differed considerably, even when the same dose was employed. In most cases the pains became stronger, and the intervals shorter. In six cases, however, it was found necessary to repeat the injection once before a satisfactory increase in the strength of the labour pains was induced, and in one case a third injection was required. Hahl,[1] who records these cases, remarks that although on the whole he obtained excellent results, he thinks that some observers have over-estimated its value in uterine inertia.

In cases where large doses are given after the membranes have ruptured, there is some danger to the child's life, as the drug shortens the period between the contractions, and raises the intra-uterine pressure during this period, so that it would appear that a large dose might cause tetanic contraction of the uterus.

The action of a second injection of pituitrin on the same patient is not invariably similar in all cases. Sometimes it acts more powerfully, and sometimes less so than the first injection. *Post partum* the drug should be given in one large dose, 1 gm. giving very satisfactory results. No toxic effects have been observed after the injection of the drug either during or after labour, but its use is not advisable in cases of high blood pressure. Ross reports thirteen cases in which he obtained good results when the labour pains were flagging, and he was thus able to deliver patients in cases where forceps would otherwise have been necessary. He never noticed any harmful effect

[1] Hahl, *Brit. M. J.*, Dec. 16th, 1911.

either on the mother or child. Schiffman[1] also records a series of fourteen cases in which pituitrin was given for abortion. In three of them it had no effect in inducing the onset of pains so long as the cervix was closed, but after dilatation pituitrin was effective in two out of the three cases. Out of seven cases in which abortion was already in progress and the cervical canal was patent, the injection was effective in four, and ineffectual in three. In some cases repeated injections were successful when a single one had failed. As a result of his experience he finds that pituitrin is extremely useful in full-time pregnancy as a means of inducing strong labour pains, especially where pains have already set in, but have become weak or ceased. It is not suited for the induction of abortion. It may, however, be useful in cases of abortion after artificial dilatation of the cervix, when introduction of the hand into the uterus is, for any reason, inadvisable.

**Obscure symptoms caused by fibromyomata of the uterus.**—There is now a considerable amount of evidence in support of the view that various obscure toxic symptoms may result from latent necrosis of a fibromyoma of the uterus. These symptoms include a rise of temperature, rapid pulse, with general and progressive debility, while beyond the presence of the tumour there may be no sign or symptom referred to the pelvic organs. In these cases careful observation of the patient over a considerable period is often necessary to decide whether the symptoms of general toxæmia are due to an inflamed condition in the pelvic organs or elsewhere. As the signs of toxæmia often include adventitious murmurs heard over the region of the heart, the physician is liable to conclude that the cardiac condition is primary and not secondary. One valuable guide to the existence of latent necrosis of a fibromyoma of the uterus is its rapid growth in the absence of pain or tenderness on pressure. Kaarsberg[2] records a series of cases in which various circulatory disturbances such as irregular action of the heart, adventitious murmurs, and cardiac dilatation appeared at an early stage, the result, he thinks, of toxæmia caused by obscure changes taking place in the tumours, some of which showed on careful examination, after removal, nothing beyond a necrotic area in the substance of the tumour. Further evidence in support of the view that these tumours may cause toxic symptoms even without the presence of necrosis is to be found in the fact that removal of the tumour has a striking influence on the health of the patient, whose vitality is markedly increased after the operation.

D. C. RAYNER.

[1] Schiffman, *Wien. klin. Wchnschr.*, No. 43, 1911.
[2] Kaarsberg, *Hospitalstidende*, Aug. 23rd, 1911.

